# Depressive Symptoms, Socioeconomic Position, and Mortality in Older People Living With and Beyond Cancer

**DOI:** 10.1097/PSY.0000000000001294

**Published:** 2024-03-18

**Authors:** Natalie Ella Miller, Abigail Fisher, Philipp Frank, Phillippa Lally, Andrew Steptoe

**Affiliations:** From the Department of Behavioural Science and Health, Institute of Epidemiology and Health Care (Miller, Fisher, Steptoe), and UCL Brain Sciences (Frank), University College London, London; and Department of Psychological Sciences (Lally), University of Surrey, Guildford, Surrey, UK.

**Keywords:** depressive symptoms, socioeconomic position, Cancer, psycho-oncology, **CES-D** = Center for Epidemiologic Studies Depression Scale, **CI** = confidence interval, **ELSA** = English Longitudinal Study for Aging, **HR** = hazard ratio, **HSE** = Health Survey for England, **LWBC** = living with and beyond cancer, **SD** = standard deviation, **SEP** = socioeconomic position, **SHR** = subdistribution hazard ratios

## Abstract

**Objective:**

Evidence shows that higher depressive symptoms are associated with mortality among people living with and beyond cancer (LWBC). However, prior studies have not accounted for a wider range of potential confounders, and no study has explored whether socioeconomic position (SEP) moderates the association. This study aimed to examine the association between depressive symptoms and mortality among people LWBC, and moderation by SEP.

**Methods:**

Participants from the English Longitudinal Study of Aging, diagnosed with cancer and with a measure of depressive symptoms within 4 years after their diagnosis, were included. Elevated depressive symptoms were indicated by a score of ≥3 on the eight-item Center for Epidemiologic Studies Depression Scale. Cox regression models examined associations with all-cause mortality. Competing risk regression examined associations with cancer mortality.

**Results:**

In 1352 people LWBC (mean age = 69.6 years), elevated depressive symptoms were associated with a 93% increased risk of all-cause mortality (95% confidence interval = 1.52–2.45) within the first 4 years of follow-up and a 48% increased risk within a 4- to 8-year follow-up (95% confidence interval = 1.02–2.13) after multivariable adjustment. Elevated depressive symptoms were associated with a 38% increased risk of cancer mortality, but not after excluding people who died within 1 year after baseline assessments. There were no interactions between depressive symptoms and SEP.

**Conclusions:**

Elevated depressive symptoms are associated with a greater risk of all-cause mortality among people LWBC within an 8-year follow-up period. Associations between depressive symptoms and cancer mortality might be due to reverse causality.

## INTRODUCTION

The number of people diagnosed with cancer is rising globally (1). Cancer survival rates are also increasing for most cancer types, due to earlier detection and advancements in treatment (2). Consequently, the number of people living with and beyond cancer (LWBC) in the UK is expected to rise by approximately one million per decade from 2010 to 2040 (3). This highlights the need to identify psychosocial factors that affect cancer outcomes among people LWBC, and ultimately to develop interventions to address these.

A cancer diagnosis and subsequent treatment can contribute to significant emotional distress (4). Cancer is commonly perceived as a life-threatening and traumatic illness that is associated with perceptions of suffering, uncertainty and mortality (5). A cancer diagnosis can evoke feelings of vulnerability, loss of control, hopelessness, fears of recurrence and mortality, body image concerns, stigma, shame, and blame (6–9). The prevalence of depression is high among people LWBC. A cross-sectional study of 10,153 people with cancer found that the prevalence of clinical depression was 13%, and that of subclinical levels was 17% (10). Case-control comparisons of 4020 people with cancer versus 5018 without found that the odds of being depressed were more than five times greater in those with cancer (11).

Higher levels of depression are related to poorer survival in people LWBC. A meta-analysis of observational cohorts found that greater symptoms of depression and anxiety were associated with an 18% increased risk of cancer-specific mortality (10 studies) and a 23% greater risk of all-cause mortality in people with cancer (15 studies) (12). Furthermore, clinically diagnosed depression and anxiety disorders were associated with a 24% increased risk of cancer-specific mortality (6 studies) and a 26% increased risk of all-cause mortality in people with cancer (5 studies). However, most studies in this meta-analysis had not controlled for all potential confounders, such as antidepressant use. Antidepressant use may affect levels of depressive symptoms and may also increase the risk of mortality (13). A more recent study (not included in the meta-analysis) of 19,966 patients with common cancers found that greater depressive symptoms were associated with poorer survival, independent of anxiety (14). However, this study did not control for comorbidities and only explored all-cause mortality. Several studies have examined associations between depressive symptoms and cancer-specific mortality in people LWBC (15–17), but none of these have accounted for competing causes of death that may have precluded death from cancers.

Although there is some evidence for a link between depressive symptoms and survival among people LWBC, it remains unclear whether there are specific sociodemographic groups who are more “at risk” and therefore in need of more targeted intervention. There are socioeconomic gradients to psychological distress in cancer, with many studies showing that people of lower socioeconomic position (SEP) experience greater psychological distress (18,19). Evidence also shows that individuals of a lower SEP have a greater risk of cancer morbidity and mortality (20,21). Given that SEP is associated with both psychological distress and survival among people LWBC, it is plausible that SEP might moderate associations between the two. Although some evidence in the general population has suggested SEP augmented the effects of psychological distress on all-cause and cardiovascular disease mortality (22,23), this has yet not been explored in people LWBC.

Therefore, the aims of this study were to examine the associations between depressive symptoms and both cancer and all-cause mortality in people LWBC, adjusting for multiple confounders. Additionally, we investigated whether SEP moderated the associations of depressive symptoms with cancer and all-cause mortality.

## METHODS

### Participants

Participants were drawn from the English Longitudinal Study of Aging (ELSA), a population-based, prospective cohort of adults 50 years and older from England (24). The sample was recruited from households that participated in the Health Survey for England (HSE) in 1998, 1999, and 2001 (Wave 0). The initial sample included 12,099 participants and was representative of the general English population. Data have been collected biennially from ELSA participants using interviews and self-completion questionnaires on topics such as physical and mental health, social and psychological factors, and behavior. Ethical approval for all ELSA waves has been obtained from the National Research and Ethics Committee, and all participants gave written informed consent before participation.

This study included ELSA participants from Waves 0–7 (1998–2015) who had been diagnosed with cancer and had a measure of depressive symptoms within 4 years after their cancer diagnosis. This time frame was selected based on prior work looking at the association between depression and cancer survival (15) to ensure a large enough sample size for survival analysis. Participants with in situ neoplasms were excluded from the sample, as these neoplasms are not always considered as cancer and are usually not treated (25). The first postdiagnosis measure of depressive symptoms represents the study baseline for each participant.

### Measurement of Depressive Symptoms

Depressive symptoms were assessed between Waves 1 and 7 using the eight-item version of the Center for Epidemiologic Studies Depression Scale (CES-D) (26). The CES-D captures information on symptoms of depression during the past week. Items ask if individuals have experienced depression, effort in everyday life, restless sleep, happiness/enjoyment in life, loneliness, sadness, and low energy levels. Each item has a yes/no response option (the scoring for positive items is reversed), resulting in a total score ranging from 0 (no symptoms) and 8 (all eight symptoms). A binary variable was constructed using the established cutoff of 3 or more to indicate elevated depressive symptoms (27). This cutoff has been shown to correspond with the traditional cutoff of 16 points on the 20-item CES-D to indicate a clinical diagnosis of depression (26,28). The CES-D has been shown to have good internal consistency at each wave of ELSA (Cronbach’s *α* > .95) (29), and the eight-item version has similar psychometric properties to the full 20-item CES-D (26). The CES-D has also been shown to have good psychometric properties in people diagnosed with cancer (27,30).

### Ascertainment of SEP

Total nonpension household wealth was defined as financial wealth (savings and investments), housing/property wealth (less mortgage), and physical wealth (e.g., land, business wealth, and jewelry) minus any debt. Wealth was categorized into tertiles for this study to enable sufficient sample sizes in each group for survival analysis.

### Cancer Characteristics

Participants were identified as having a cancer diagnosis using cancer data supplied by NHS Digital on behalf of Public Health England. All cancer types were denoted by *International Classification of Disease, Ninth Edition* (*ICD-9*) (31) and *Tenth Edition* (*ICD-10*) (32) codes. Categories of cancers related to smoking and alcohol were devised based on the *ICD-10*. Date of diagnosis was used to calculate age at diagnosis and time between diagnosis and baseline assessments. Because ELSA was not set up to study cancer, information on cancer stage or treatments was not requested from cancer registry and was therefore not available for analyses. Self-report data on whether participants received treatment or not were available, but only in the past 2 years, so these data were not analyzed.

### Measurement of Covariates

In this study, we adjusted analyses for relevant sociodemograpic and health-related covariates based on prior research exploring the association between depression and survival in people LWBC (14,15). All covariates were measured at baseline. These covariates included age, sex (male/female), ethnicity (White/non-White), marital status (married/not married), wealth tertiles, number of comorbidities, cancer being smoking related/not, cancer being alcohol related/not, days between baseline assessments and cancer diagnosis, age at cancer diagnosis, and antidepressant use (yes/no). The number of comorbidities was assessed by self-reported doctor diagnosed chronic diseases, including arthritis, asthma, Alzheimer’s disease, dementia, lung disease, Parkinson’s disease, and cardiovascular diseases (diabetes, high blood pressure, angina, heart attack, stroke, heart failure, heart murmur or heart rhythm). Alcohol-related cancers were defined as established cancers of the breast, colorectum, liver, esophagus, and oral cavity and pharynx, according to the National Cancer Institute (33). Smoking-related cancers were defined as established cancers of the cervix and female reproductive system, colorectum, kidney, liver, esophagus, oral cavity and pharynx, pancreas, respiratory system, stomach, urinary system, and leukemia, according to the National Cancer Institute (34). Antidepressant use was recorded at Wave 0 and included either selective serotonin reuptake inhibitors or tricyclic antidepressants.

### Ascertainment of All-Cause and Cause-Specific Mortality

Mortality data (data and cause of death) spanning from baseline to Wave 9 (2018–2019) were obtained from NHS Digital on behalf of the Office for National Statistics. Causes of mortality were classified according to the *ICD-10* (32) and categorized into cancer and all-cause mortality.

### Statistical Analysis

#### Missing Data

To reduce bias caused by missing data, multiple imputation by chained equations was used to impute missing information on the covariates (35). We included auxiliary variables associated with nonresponse in ELSA: highest level of education, year of Health Survey for England interview, and household size. Twenty imputed datasets were generated and pooled using Rubin’s rules (36).

#### Descriptive Statistics

Descriptive statistics for both the observed and imputed datasets were first calculated. Means and standard deviations were calculated for continuous variables, and frequencies and percentages were computed for categorical variables.

#### Associations Between Depressive Symptoms and Mortality

We performed a series of adjusted Cox proportional hazards models to examine the prospective association between depressive symptoms (high/low) and all-cause mortality. Competing-risk regression based on Fine and Gray’s proportional subhazard model (37) was used for cancer mortality, as this method takes into account competing events that may prevent the event of interest from occurring. Hazard ratios (HRs) and their 95% confidence intervals (CIs) were calculated for Cox proportional hazards regression. Subdistribution HRs and their 95% CIs were calculated for competing-risk regression. Survival time was measured in years from time of baseline assessments to date of death, or to end of follow-up (March 2019). Effect estimates were adjusted for a) age and sex, and b) 1 + sociodemographic factors, comorbidities, cancer characteristics, and antidepressant use. To assess interactions between SEP (indexed by wealth) and depressive symptoms, an interaction term was added to the fully adjusted models, and the Wald test was used. Stata version 17.0 was used for all analyses.

The proportional hazards assumption was violated according to Schoenfeld residuals. Therefore, it was appropriate to use four follow-up periods to stratify the analyses (<4, 4–8, 8–12, and ≥12 years). The results from these analyses are shown in Table S1, Supplemental Digital Content, http://links.lww.com/PSYMED/B8. Because of small sample sizes and a lack of effects, the last two follow-up periods were collapsed, and the final analyses were stratified by three follow-up periods (<4, 4–8, and ≥8 years). Multicollinearity was tested and ruled out.

#### Sensitivity Analyses

Several sensitivity analyses were conducted. First, participants were excluded if they died within 1 year of baseline assessments of depression to reduce the risk of reverse causality (whereby more advanced disease status could confound associations by increasing the risk of dying and depressive symptoms). Second, analyses were run on a sample of participants with no missing data on the exposure, outcome, and covariates. Third, depressive symptoms were entered as a continuous rather than binary variable.

## RESULTS

### Descriptive Statistics

Table 1 reports baseline characteristics of the analytical sample (see Table S2, Supplemental Digital Content, http://links.lww.com/PSYMED/B8, for a comparison of baseline characteristics in the observed versus imputed data). Of 1352 included participants, average age at baseline was 69.6 years (51.5% male). Approximately one quarter of the sample reported elevated depressive symptoms (26.1%). A third of the sample were in the lowest wealth quintile (mean wealth = £44,820), a third were in the middle wealth quintile (mean wealth = £209,100), and a third were in the highest wealth quintile (mean wealth = £616,524). During a mean follow-up period of 7.3 years (range: 0–16 years), a total of 596 (44.1%) participants died, of which 314 (52.7%) died within 4 years of their baseline assessments, 154 (25.8%) between 4 and 8 years of their baseline assessments, and 128 (21.5%) between 8 and 16 years of their baseline assessments. Of the 596 deaths that occurred, 335 (28.4%) were from cancer.

**TABLE 1:** Participant Characteristics at Baseline

	Total (*N* = 1352)	Died (*N* = 596)	Alive (*N* = 756)
	Mean (SD)/*n* (%)*^a^*	Mean (SD)/*n* (%)*^a^*	Mean (SD)/*n* (%)*^a^*
Age, y	69.6 (12.7)	71.8 (15.8)	67.9 (9.26)
Sex			
Male	696 (51.5)	323 (54.2)	373 (49.3)
Female	656 (48.5)	273 (45.8)	383 (50.7)
Ethnicity			
White	1328 (98.2)	588 (98.6)	744 (98.4)
Non-White	24 (1.8)	8 (1.4)	12 (1.6)
Marital status			
Married	867 (64.1)	347 (58.2)	520 (68.8)
Not married	485 (35.9)	249 (41.8)	236 (31.2)
Wealth tertile			
1 (least wealthy)	450 (33.3)	245 (41.2)	202 (26.7)
2	450 (33.3)	200 (33.5)	253 (33.4)
3 (wealthiest)	452 (33.4)	151 (25.3)	301 (39.8)
Total number of comorbidities	1.86 (1.50)	2.14 (1.56)	1.64 (1.41)
0	238 (17.6)	70 (11.7)	168 (22.2)
1	388 (28.7)	166 (27.9)	222 (29.4)
2	358 (26.5)	158 (26.5)	200 (26.5)
3	193 (14.3)	97 (16.3)	96 (12.7)
4	97 (7.2)	56 (9.4)	41 (5.4)
5	41 (3.0)	27 (4.5)	14 (1.9)
6+	37 (2.8)	22 (3.7)	15 (1.9)
Cancer type			
Head and neck	23 (1.7)	12 (2.0)	11 (1.5)
Colorectal	128 (9.5)	62 (10.4)	66 (8.7)
Other gastrointestinal cancers (excluding colorectal)	34 (2.5)	20 (3.4)	14 (1.9)
Respiratory system	58 (4.3)	45 (7.6)	13 (1.7)
Melanoma	38 (2.8)	14 (2.3)	24 (3.2)
Other skin neoplasms (excluding melanoma)	423 (31.3)	148 (24.8)	275 (36.4)
Breast	179 (13.2)	58 (9.7)	121 (16.1)
Female reproductive	65 (4.8)	32 (5.34	33 (4.4)
Male reproductive	195 (14.4)	86 (14.4)	109 (14.4)
Urinary system	64 (4.7)	37 (6.2)	27 (3.6)
Hemotological	76 (5.6)	44 (7.4)	32 (4.2)
Other*^b^*	69 (5.1)	38 (6.4)	31 (4.1)
Smoking-related cancer			
Yes	316 (23.4)	187 (31.4)	129 (17.1)
No	1036 (76.6)	409 (68.6)	627 (82.9)
Alcohol-related cancer			
Yes	345 (25.5)	139 (23.3)	206 (27.2)
No	1007 (74.5)	457 (76.7)	550 (72.8)
Age at diagnosis, y	70.5 (9.4)	74.2 (9.0)	67.6 (8.5)
Time between baseline assessments and cancer diagnosis, d	447.5 (323.2)	473.0 (333.5)	427.3 (313.7)
Antidepressant use			
Yes	65 (4.8)	29 (4.9)	35 (4.6)
No	1287 (95.2)	567 (95.1)	721 (95.4)
Depressive symptoms severity score	1.7 (2.0)	2.10 (2.10)	1.40 (1.90)
High depressive symptoms	353 (26.1)	205 (34.4)	148 (19.6)
Low depressive symptoms	999 (73.4)	391 (65.6)	608 (80.4)

SD = standard deviation.

Values are presented as means (SD) for continuous variables and *n* (%) for categorical variables.

*^a^* Pooled *n* values and % are shown across the 20 imputed datasets.

^*b*^ Includes cancers of uncertain/unknown behavior; connective, subcutaneous, and other soft tissue; Kaposi; brain; cranial nerves and other nervous system; eye and adnexa; thyroid and other endocrine glands; and ill defined, secondary and unspecified sites.

Of the 353 participants who reported elevated depressive symptoms, 205 (58.1%) died over the follow-up period. Of the 999 participants who did not report elevated depressive symptoms, 391 (39.1%) died over the follow-up period. This corresponded to 98.5 deaths per 1000 person years among those who reported elevated depressive symptoms, and 50.6 deaths per 1000 person years among those who did not report elevated depressive symptoms (% absolute excess risk = 4.8).

### Association of Depressive Symptoms With All-Cause Mortality

In analyses of the follow-up period of less than 4 years, high depressive symptoms (versus low) were associated with greater risk of all-cause mortality after adjustment for age and sex (HR = 2.35; 95% CI = 1.87–2.95) (Table 2). High depressive symptoms were associated with 93% increased risk for all-cause mortality after further adjustment for ethnic origin, marital status, wealth, number of comorbidities, cancer characteristics, and antidepressant use (95% CI = 1.52–2.45).

**TABLE 2:** Association Between High Depressive Symptoms and All-Cause Mortality Risk Over 16.1 Years of Follow-Up Among People LWBC

	All-Cause Mortality	
	HR (95% CI)	*P*
<4 y	*N* deaths/*N* total = 314/1352	
Model 1	2.35 (1.87–2.95)	<0.001***
Model 2	1.93 (1.52–2.45)	<0.001***
4–8 y	*N* deaths/*N* total = 154/919	
Model 1	1.74 (1.23–2.46)	0.002**
Model 2	1.48 (1.02–2.13)	0.037*
≥8 y	*N* deaths/*N* total = 128/509	
Model 1	1.12 (0.72–1.74)	0.621
Model 2	1.07 (0.67–1.70)	0.777

LWBC = living with and beyond cancer; HR = hazard ratio; CI = confidence interval.

Low depressive symptoms = reference category. Model 1 adjusted for age and sex. Model 2 adjusted for age, sex, ethnicity, marital status, wealth, number of comorbidities, alcohol-related cancer type (yes/no), smoking-related cancer type (yes/no), age at cancer diagnosis, time between cancer diagnosis and depressive symptoms assessment, and antidepressant medication (yes/no)

* *p* < .05.

** *p* < .01.

*** *p* < .001.

When the follow-up period was between 4 and 8 years, high depressive symptoms (versus low) were associated with a 74% greater risk of all-cause mortality after adjustment for age and sex (95% CI = 1.23–2.46) and a 48% increased risk of all-cause mortality after further adjustment for ethnic origin, marital status, wealth, number of comorbidities, cancer characteristics, and antidepressant use (95% CI = 1.02–2.13).

When the follow-up period was 8 years or more, there was no statistically significant association at conventional levels between high/low depressive symptoms in any model (*p* > .05). The full details of the final regression results for the above analyses are shown in Table S3, Supplemental Digital Content, http://links.lww.com/PSYMED/B8. Other predictors of all-cause mortality were ethnicity, smoking-related cancer/not, alcohol-related cancer/not, and age at diagnosis when follow-up was less than 4 years; number of comorbidities, smoking-related cancer/not, and age at diagnosis when follow-up was 4 to 8 years; and marital status, days between baseline assessments and cancer diagnosis, age at diagnosis, and antidepressant use when follow-up was 8 years or more.

#### Interaction Between Depressive Symptoms and SEP

There was no statistically significant interaction between depressive symptoms and wealth in the prediction of all-cause mortality when the follow-up period was less than 4 years (*p* = .17), between 4 and 8 years (*p* = .18), and 8 years or more (*p* = .79).

### Association of Depressive Symptoms With Cancer Mortality

After adjustment for age and sex, elevated depressive symptoms (versus low) were associated with a 42% greater risk of cancer mortality (95% CI = 1.12–1.81) (Table 3). High depressive symptoms were also associated with a 38% greater risk of cancer mortality after further adjustment for ethnic origin, marital status, wealth, number of comorbidities, cancer characteristics, and antidepressant use (95% CI = 1.07–1.78). Full details of the final regression results are shown in Table S4, Supplemental Digital Content, http://links.lww.com/PSYMED/B8. Other predictors of cancer mortality were smoking-related cancer/not, alcohol-related cancer/not, and age at diagnosis.

**TABLE 3:** Association Between High Depressive Symptoms and Cancer-Specific Mortality Risk Over 16.1 Years of Follow-Up Among People LWBC

	Cancer-Specific Mortality	
	SHR (95% CI)	*P*
	*N* Deaths/*N* Total = 335/1352	
Model 1	1.42 (1.12–1.81)	0.004**
Model 2	1.38 (1.07–1.78)	0.014*

LWBC = living with and beyond cancer; SHR = subdistribution hazard ratio; CI = confidence interval.

Low depressive symptoms = reference category. Model 1 adjusted for age and sex. Model 2 adjusted for age, sex, ethnicity, marital status, wealth, number of comorbidities, alcohol-related cancer type (yes/no), smoking-related cancer type (yes/no), age at cancer diagnosis, time between cancer diagnosis and depressive symptoms assessment, and antidepressant medication (yes/no).

* *p* < .05.

** *p* < .01.

#### Interaction Between Depressive Symptoms and SEP

There was no statistically significant interaction between depressive symptoms and wealth in the prediction of cancer mortality (*p* = .73).

### Sensitivity Analyses

The first sensitivity analysis tested the association between high/low depressive symptoms and mortality risk, excluding participants who died within 1 year of baseline assessments. For all-cause mortality, the results were similar to those observed in the main analyses (*N* = 1246 for <4 years; *N* = 919 for 4–8 years; *N* = 509 for ≥8 years) (Table S5, Supplemental Digital Content, http://links.lww.com/PSYMED/B8). However, there was no association between depressive symptoms and cancer mortality in any model (*N* = 1246) (Table S6).

In the second sensitivity analysis, we tested the association between high/low depressive symptoms and mortality risk in participants with no missing data on the exposure, outcome, and covariates. For all-cause mortality, results were similar to the main analyses when the follow-up duration was less than 4 years (*N* = 1159) and 8 years or more (*N* = 772) (Table S7, http://links.lww.com/PSYMED/B8). However, when the follow-up duration was 4 to 8 years, there was no statistically significant association between depressive symptoms and all-cause mortality after multivariable adjustment (Model 2; *p* = .05; *N* = 391). For cancer mortality, results were similar to those of the main analyses (*N* = 1159) (Table S8).

The third sensitivity analysis tested the association between depressive symptoms (continuous variable) and mortality risk. The results for all-cause mortality were similar to the main analyses (*N* = 1352 for <4 years; *N* = 919 for 4–8 years; *N* = 509 for ≥8 years) (Table S9, Supplemental Digital Content, http://links.lww.com/PSYMED/B8). However, when the follow-up duration was 4 to 8 years, the findings were directionally consistent with associations found in the main analysis but did not reach statistical significance at conventional levels (Model 2; *p* = .30). For cancer mortality, the results closely mirrored those found in the main analyses (*N* = 1352) (Table S10).

## DISCUSSION

In this study of older people LWBC, high depressive symptoms were associated with increased risk for all-cause mortality when the follow-up period was less than 8 years. This association remained significant after adjusting for number of comorbidities, cancer-related characteristics, and antidepressant use, suggesting that the association is independent of these factors. The association was stronger when the follow-up period was shorter (less than 4 years compared to between 4 and 8 years). High depressive symptoms were also associated with increased risk for cancer mortality. However, this association disappeared after excluding people who died within a year after baseline assessments, suggesting that reverse causality may have contributed to the association. SEP did not moderate any association between depressive symptoms and mortality.

High depressive symptoms were associated with increased risk for all-cause mortality in people LWBC when the follow-up period was less than 8 years. This association was even stronger when the follow-up was less than 4 years compared to between 4 and 8 years. However, no association was found during a 4-to 8-year follow-up period when participants with no missing data on the exposure, outcome, and covariates were included, perhaps due to a lack of power given the smaller sample size. Nevertheless, all our analyses met the conventional threshold for an adequate sample size for Cox regression, which is 10 events per variable (38,39). Again, no association was found during a 4- to 8-year follow-up period when a continuous measure of depressive symptoms was used, suggesting that having one or two depressive symptoms may not be important, but that the presence of several symptoms is prognostic. No association between depressive symptoms and all-cause mortality was found when the follow-up period was 8 years or more. These findings are all in line with prior reviews showing that studies finding a positive association between depression and mortality in people LWBC tend to have a shorter-follow-up, whereas studies finding no association tend to have a longer follow-up (40,41). A meta-analysis found that of 20 studies looking at the association between depression and anxiety and risk of all-cause mortality in people LWBC, studies with longer follow-up (≥10 years) had lower risk ratios (1.06 [95% CI = 1.00–1.06] versus 1.36 [95% CI = 1.21–1.52]) than studies with shorter follow-up (<10 years) (12). However, most studies in this meta-analysis did not control for antidepressant use. Our study showed that the association between high depressive symptoms and greater risk for all-cause mortality in shorter follow-up periods was independent of many potential confounders, including antidepressant use. The magnitude of the effect estimates found in this study (93% increased risk for less than 4-year follow-up and 48% increased risk for 4- to 8-year follow-up) is greater than for those found in prior studies with follow-up of less than 10 years (12), perhaps due to even shorter follow-up.

One reason why the association between high depressive symptoms and risk of all-cause mortality is only found in shorter follow-up periods is that longer follow-ups may reflect less severe or more treatable cancer. Individuals with less severe disease may also experience less fear of recurrence. In our study, we could not adjust for measures of cancer severity like cancer stage or treatment due to lack of available data. Another reason is that severity of depressive symptoms may change over time, meaning that baseline scores may have less predictive power with longer follow-up. Studies have shown that depression decreases in severity over time after a cancer diagnosis (42), and hence, the strength of the association with mortality may weaken as a result. Furthermore, as follow-up increases, other intervening health factors (e.g., new illnesses/treatments) may interfere with the association between depression and mortality (43). Future work could examine associations between trajectories of depressive symptoms and mortality in people LWBC to account for the effect of follow-up duration on the association.

There are several, albeit understudied, mechanisms that might explain the association between high depressive symptoms and all-cause mortality in people LWBC. First, health behaviors may explain the association. There is evidence that higher depressive symptoms are associated with unhealthier behaviors (lower physical activity, greater alcohol consumption and smoking) in people LWBC (44). Furthermore, unhealthier behaviors are associated with increased risk for mortality in people LWBC (45). Second, adherence to medical treatments and appointments may contribute to the association. There is evidence that people LWBC with depression are less likely to attend medical appointments (46) and have treatments like chemotherapy (47), which may, in turn, increase risk for mortality. Third, biological factors like inflammation might mediate the association between depressive symptoms and mortality in people LWBC. Numerous population-based studies have found prospective associations between depression and biomarkers of systemic inflammation such as C-reactive protein (48,49). Inflammation has also been associated with poorer cancer survival in several prospective, population-based studies (50–52). A complex pathway might exist between depressive symptoms, health behaviors, inflammation, and survival in people LWBC, as evidence shows that health behaviors such as physical activity partly mediate the association between depressive symptoms and elevated inflammation in the general population (53). Future work is needed to unravel the mechanisms linking high depressive symptoms and all-cause mortality in people LWBC.

Our study also showed that high depressive symptoms were associated with increased risk for cancer mortality, but not after excluding participants who died within a year after baseline assessments. This finding suggests that the association between depressive symptoms and cancer mortality may be due to reverse causality, whereby cancer progression can also influence depression. Cancer progression may influence depression through physiological mechanisms, the psychological impact of expecting a rapid decline, or as a result of the functional limitations associated with progressing cancer. Alternatively, it is possible that people who are most highly depressed die most rapidly, which might explain the importance of the <1-year mortality group. Some prior studies that have found associations between depression and cancer mortality have not accounted for reverse causality (15,54). A meta-analysis found associations between depressive symptoms and cancer mortality even after accounting for reverse causality (12). However, most studies in this meta-analysis used Cox proportional hazards models to analyze associations between depressive symptoms and cancer mortality and did not account for competing causes of death. By not accounting for competing causes of death, prior studies might have overestimated the association between depressive symptoms and cancer mortality. Furthermore, this meta-analysis had a larger sample size than the present study and thus might have been more powered to detect an association. Future work replicating this study in larger samples, using competing risk regression, is needed to confirm whether an association between depressive symptoms and cancer mortality exists, or whether it is due to reverse causality.

SEP did not moderate any associations between depressive symptoms and mortality in this study. This finding was surprising given the socioeconomic gradients in depression (18,19) and mortality rates (21,55) in people LWBC. People of higher SEP tend to have greater economic, social and psychological resources to cope with stress. Prior studies have found that SEP augments the effects of psychological distress on all-cause and cardiovascular disease mortality rates in the general population (22,23). However, these prior studies used occupational class as an indicator of SEP, whereas our study used wealth. We used wealth because it is the most robust indicator of socioeconomic circumstances in ELSA and is more strongly associated with risk of mortality than any other SEP indicator at older ages (56). The use of occupational class to determine SEP is problematic in older people who tend to be retired (57). Another reason for our discrepant findings is that both studies by Lazzarino and colleagues used the 12-item General Health Questionnaire to capture symptoms of anxiety, depression, and general psychological distress, whereas our study used the CES-D to capture depressive symptoms only. It is a strength that our study considered depression independently, given that a study of 19,666 people with common cancers found that depression and anxiety have different independent associations with survival in people with cancer (14). Alternatively, it is possible that our sample was not powered enough to detect an interaction between depressive symptoms and SEP in predicting mortality.

There are several strengths of the current study. This study a) accounted for a wide array of confounders, b) used a long follow-up to test the association between depressive symptoms and mortality, c) applied data linkage to national cancer registry and mortality data, and d) accounted for missing data using multiple imputation.

However, this study also had some limitations. First, we could not adjust for measures of cancer severity such as cancer stage and treatment due to lack of available data. Second, the measure of depressive symptoms used (CES-D) does not capture specific elements relating to a cancer diagnosis. Furthermore, symptoms of depression like low mood and fatigue can overlap with the physical symptoms caused by a cancer diagnosis. Nevertheless, the CES-D captures cognitive and affective elements of depression, rather than physical issues, and thus is useful for medically ill populations like people LWBC (30). Third, participants had an average 1-year gap between their cancer diagnosis and baseline assessments of depression, meaning that their depression may have been due to factors other than their diagnosis, and that peak levels of depression occurring soon after diagnosis might have been missed. However, cancer is associated with increased rates of depression across various types, as shown in a meta-analysis of over 200 studies (58). Another study of 1966 people affected by colorectal cancer found that a third of the sample experienced distress 5 years after diagnosis (59). Thus, it is likely that at least some of the depression measured in this study was due to cancer. We controlled for time between diagnosis and baseline assessments of depression to adjust for any effects on the association between depression and survival. Fourth, this study combined different cancer types, even though cancer is a heterogeneous condition. Combining different cancer types may mask associations between depressive symptoms and mortality for certain cancers. Future work could replicate this study in specific cancer types to untangle site-specific associations. Fifth, all participants were older people, which limits generalization to younger individuals, especially given that the association between depression and mortality has been shown to be stronger in older than younger people LWBC (43).

In conclusion, this study shows that high depressive symptoms are associated with a greater risk for all-cause mortality among people LWBC, particularly within the first 8 years after diagnosis. Our finding highlights the need to screen for and effectively treat depression after a cancer diagnosis. Particular attention should be paid to the early phases of diagnosis when the implications of being more depressed are greatest. Future work is needed to identify the underlying mechanisms and effective interventions to screen for and treat depression in people LWBC.
